# Detection of bacteria in middle ear effusions based on the presence of allergy: does allergy augment bacterial infection in the middle ear?

**DOI:** 10.1186/s40463-015-0111-5

**Published:** 2015-12-29

**Authors:** Woo Jin Kim, Byung-Guk Kim, Ki-Hong Chang, Jeong-Hoon Oh

**Affiliations:** Department of Otorhinolaryngology-Head and Neck Surgery, College of Medicine, The Catholic University of Korea, Seoul, Korea; Department of Otorhinolaryngology-Head and Neck Surgery, The Catholic University of Korea, 180 Wangsan-Ro, Dongdaemun-Gu, Seoul, 130-709 South Korea

**Keywords:** Middle ear effusion, Bacteria, Allergy

## Abstract

**Background:**

Bacterial infection, Eustachian tube dysfunction, allergies, and immunologic factors are major causes of otitis media with effusion (OME). However, the exact pathogenesis of OME is still unclear. This study evaluated whether allergy influences bacterial growth in middle ear effusions.

**Materials:**

Fifty-four samples were obtained from OME patients 3–10 years of age who underwent ventilation tube insertion and were divided into two groups based on the presence of allergy as determined using the multiple allergosorbent test (MAST). *Streptococcus pneumoniae*, *Haemophilus influenzae*, and *Moraxella catarrhalis* bacterial DNA in the middle ear effusions was analyzed using polymerase chain reaction. Overall detection rates and those for each species were compared between the two groups.

**Results:**

Of the 54 middle ear effusion samples, 38 (70.4 %) contained bacterial DNA and 14 (36.8 %) of these contained DNA from multiple species. *S. pneumoniae* was detected in 27 samples (50 %), *H. influenzae* in 17 samples (31.4 %), and *M. catarrhalis* in 9 samples (16.6 %). There was no significant difference in the bacterial detection rates between the middle ear effusions of the MAST-positive and MAST-negative groups.

**Conclusion:**

The rate of bacteria detection in middle ear effusions did not differ between allergic and non-allergic children.

## Background

Otitis media with effusion (OME), fluid persisting in the middle ear cavity for more than 3 months, has been attributed to various causes [1]. Eustachian tube dysfunction is one of the most important factors in the development of this disease [2–4]. However, the exact pathogenic mechanism of OME is unclear. Eustachian tube dysfunction, including obstruction and abnormal patency, can be caused by extrinsic and intrinsic factors due to infection or allergy. The role of allergies in Eustachian tube dysfunction has been emphasized. The rate of Eustachian tube function tympanometry abnormalities in a group of allergic rhinitis patients was higher than in healthy subjects [5]. These functional abnormalities may also be related to impairment of the mucociliary activity that facilitates reflow and aspiration of bacteria from the Eustachian tube [6]. The response to combined nasal instillation of bacteria and allergen in sensitized mice was more persistent than the response to either alone [7, 8]. Therefore, we hypothesized that the local allergic response in the middle ear can augment bacterial infection, which may lead to OME.

**Fig. 1:**
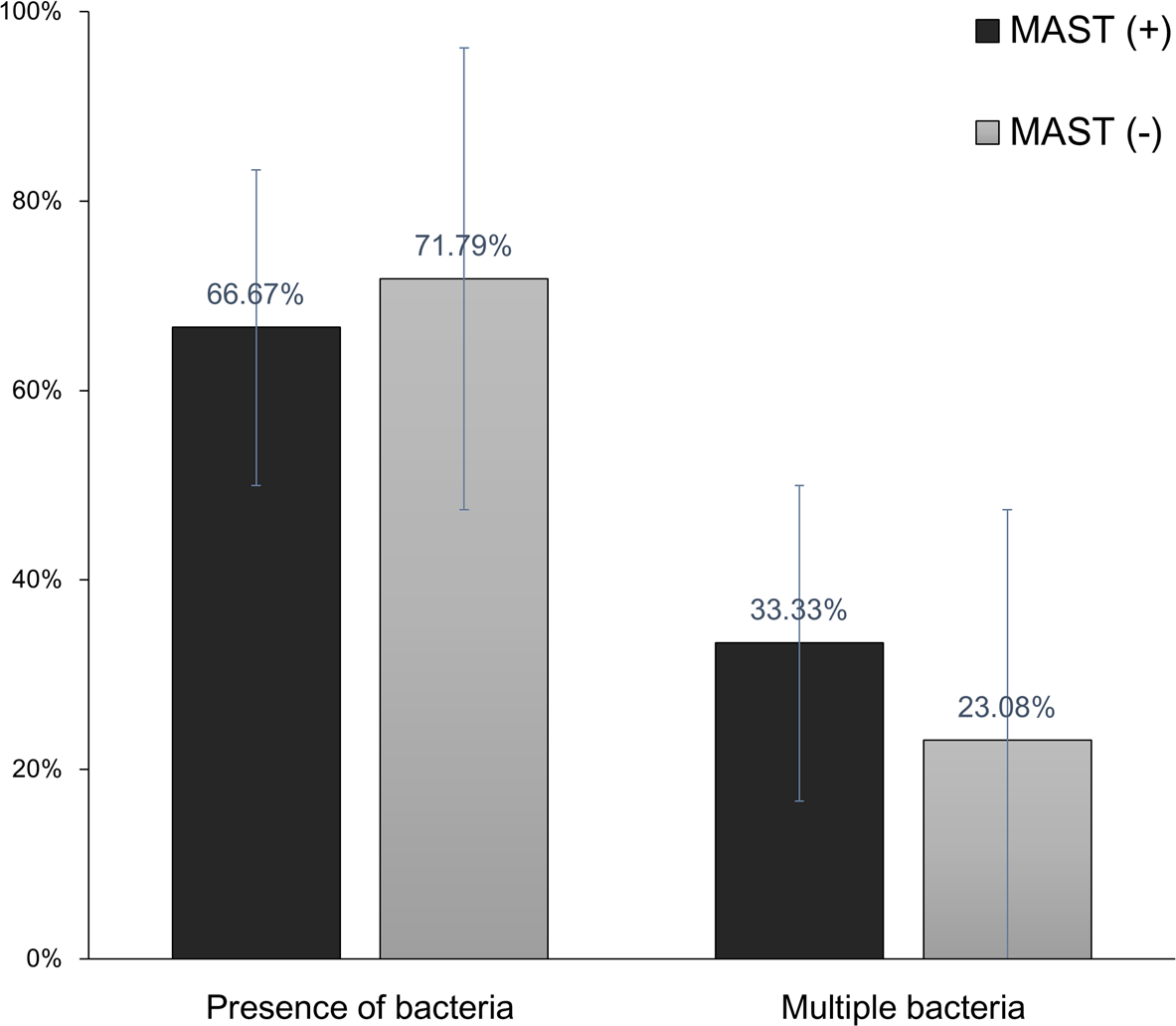
The rates of the overall detection of bacteria and detection of multiple bacteria in middle ear effusion using polymerase chain reaction in MAST-positive and -negative groups (MAST, multiple allergosorbent test)

The most common organisms that cause OME are *Streptococcus pneumoniae*, *Haemophilus influenza*, and *Moraxella catarrhalis* [2, 6, 9, 10]. Although the presence of these bacteria DNA does not mean that bacterial infection is one of the main causes of OME, there is increasing interest in the role of bacterial infection and associated inflammation as a cause of OME. Since respiratory infections and host immunity may be important in the pathophysiology of OME in children with Eustachian tube dysfunction, the presence of bacteria in OME can be affected by the patient’s immune status. Therefore, this study evaluated the correlation between allergy and bacterial infection in the middle ear of OME patients.

In this study, the presence of bacterial infection in middle ear effusion (MEE) of the children younger than 12 years old was estimated from the detection rates of the bacterial pathogens *S. pneumoniae*, *H. influenzae*, and *M. catarrhalis* using polymerase chain reaction (PCR) and conventional culture methods. Among them, the detection rates of bacteria in the MEEs of patients with allergy according to the results of the multiple allergosorbent test (MAST) were compared with the rates in patients who did not have allergy.

## Methods

This study was approved by the institutional review board and the parents provided informed consent before participation. The enrolled subjects consisted of 34 consecutive pediatric patients (19 boys, 15 girls) under 12 years old, who admitted our clinic for the insertion of ventilating tube due to OME persisting for more than 3 months. The subjects were divided into two groups according to the presence of allergy. To determine the presence of allergy, blood samples were collected before the insertion of ventilating tube for the Korean panel of the multiple allergosorbent test chemiluminescent assay (MAST-CLA) (MAST Immunosystems, Mountain View, CA, USA). This assay consists of 35 different specific IgE antibodies with associated allergens from food, mold, pollen, and inhalant allergens that are most frequently positive in Koreans. The MAST-CLA was performed according to the' manufacturer's instructions. The amount of the produced chemiluminescence, which is proportional to the amount of allergen-specific IgE in the test serum, was measured in a densitometer; the results were interpreted as classes 0, 0/1, 1, 2, 3, or 4 based on the amount of light emitted, with classes 2 to 4 considered positive results. At the time of tube placement surgery, the external auditory canal was irrigated with 70 % alcohol and then the middle ear fluid was collected using a suction collector (Storz®, Germany). The collected fluid was stored immediately at −70 °C for subsequent analysis. The genomic DNA was extracted by mixing 50 μL of the stored middle ear effusion with 900 μL of cell lysis solution, followed by a 10 min centrifugation at 15,000 rpm at room temperature. DNA was extracted using PCR premix (Bioneer®, Daejeon, South Korea). For PCR, P6 protein was used as a primer for *Haemophilus influenzae*, PBP 2B for *Streptococcus pneumoniae*, and the M46 clone for *Moraxella catarrhalis*. Thirty-five cycles of 95 °C, 55 °C, and 70 °C were performed using a DNA thermal cycler. To detect the amplified product, DNA was electrophoresed for 30 min in 2 % agarose gels. Specimens with consistent PCR results were used as positive controls and distilled water as a negative control.

The overall detection rate and that for each bacterial species was compared between the MAST-positive and -negative groups using the chi-square test.

## Results

Fifty-four ears of 34 children from 3–10 years old were enrolled in this study. Of the 54 ears, 15 were positive as determined by MAST, while the remaining 39 were negative. The mean ages of the children in the MAST-positive and -negative groups were 3.73 ± 2.25 years and 3.23 ± 0.93 years, respectively. The rate of bacteria detection using conventional culture methods was only 9.0 % (5/54) and the species cultured were *S. pneumoniae*, *S. epidermidis*, *S. capitis*, and α-hemolytic *Streptococcus*. The overall detection rate of bacterial DNA using PCR was 70.4 % (38 of 54 ears) (Table 1). In 14 of 38 ears (36.8 %), two or more bacterial species were detected in the same effusion sample. The overall detection rate of the bacteria did not differ significantly (*p* > 0.05) between the MAST-positive and -negative groups, nor did the detection rates of each bacterial species (Fig. 1). There was no significant difference in the detection rate of multiple bacteria between the two groups (Fig.1).

**Table 1:** The detection rates of major pathogenic bacteria in middle ear effusions using PCR in MAST-positive and -negative groups

Bacterial species	No (%) of PCR-positive specimens in the MAST-positive group	No (%) of PCR-positive specimens in the MAST-negative group
*S. pneumoniae*	6 (40.0 %)	21 (53.9 %)
*H. influenzae*	7 (46.7 %)	10 (25.6 %)
*M. catarrhalis*	3 (20.0 %)	6 (15.4 %)

*PCR* polymerase chain reaction; *MAST* multiple allergosorbent test

## Discussion

This study demonstrates that the children with evidence of allergy were equally likely as children without evidence of allergy to demonstrate evidence of bacterial infection in PCR tested MEE specimens. Although this study did not demonstrate a positive relationship between allergy and the bacterial infection in the pathogenesis of OME. However, numerous studies have indicated that allergic subjects are more susceptible to OME than non-allergic controls. The combination of allergic and bacterial stimulation can augment the development of OME. Labadie et al. showed that lipopolysaccharide (LPS)-induced OME, in which LPS simulated bacterial exposure, was more prominent in allergic rats [11]. Ebmeyer et al. found that the reaction to combined bacteria and allergen in sensitized mice was more persistent than the response to either alone and a reaction was absent in mast cell-deficient mice [12]. However, the exact mechanism of the relationship between the presence of bacteria in middle ear effusion and systemic immunity is still not clear. Theoretically, nasal obstruction due to an allergy can lead to an increase in negative pressure in the nasopharynx, resulting secondarily in decreased Eustachian tube patency, and disturbance of its ciliary epithelial function with subsequent increase in the negative pressure in the middle ear cavity. Several theories have been proposed to explain the Eustachian tube dysfunction caused by allergic inflammation, including retrograde spread of edema, poor mucociliary function, and venous engorgement and hypersecretion of mucus [3]. It has been suggested that the development of OME is the result of improperly functioning barriers (mucociliary system, immune system, and Eustachian tube) conferring resistance to bacteria [13]. The pathogenesis of OME is related primarily to Eustachian tube dysfunction, including obstruction, abnormal patency, and non-optimally functioning ciliated epithelium [3, 14]. These conditions can facilitate the aspiration of nasopharyngeal secretions containing pathogenic bacteria into the middle ear cavity, leading to the development of OME. The high prevalence of this condition in children reflects immaturity of function of both the immune system and the Eustachian tubes. The Eustachian tube of the infants has a smaller caliber and shorter length and joins the nasopharynx at a more acute angle in relation to the adults. All of these features predispose to dysfunction of the Eustachian tube and therefore increased risk of infection [15]. The negative finding of our study indicates that the anatomical immaturity of the Eustachian tube in children may plays a major role in the development of OME due to the bacterial infection rather than allergy. Chronic OME due to Eustachian tube dysfunction caused by allergy tends to occur much more frequently in adults than in children [3]. The analysis of differences of the detection rates among various age groups was not performed in this study. It would be helpful to distinguish the different pathogenesis of OME between children and adults.

There are several large literature reviews of the relationship of allergy to OME. Doyle concluded that allergy has been “reasonably well demonstrated” as a risk factor for otitis media.” [16]; Tewfik and Mazer determined that Th2 mediated allergic inflammation was found in MEE in patients with OME [17]. In a prospective, cohort study of patients cared for in a private community practice, all 89 OME patients proved to be atopic and specific allergy immunotherapy resolved 85 % of OME [18]. Although, these results support the hypothesis that OME is an immune mediated allergic disease, evidence for direct causal relationship between OME and allergy have been lacking. Recent study showed that the prevalence of allergic rhinitis was not different in 6- to 7-year-old children with OME and the reference group, suggesting a limited effect of allergy in the pathogenesis of OME in this age group [19]. Another study reported that no difference in Eustachian tube function was found in either the allergic rhinitis or control groups, which was interpreted as showing allergic rhinitis has little direct effect on Eustachian tube function [20].

One of the main reasons for the conflicting results regarding the relationship of allergy and OME in the literatures may due to non-controlled study designs, including diagnostic criteria for allergy and differing populations [21]. The reported incidence of allergic rhinitis in OME ranges from 14 % to as high as 89 % [17, 22]. For the diagnosis of allergy and determination of allergens, many procedures can be used: skin tests, provocation tests, the radio-allergosorbent test (RAST), and MAST-CLA. In this study, the presence of allergy was defined using the MAST-CLA system, which is used to detect allergen-specific IgE antibodies using enzyme-linked anti-human IgE and a chemiluminescent assay, as reported previously [23]. This test avoids the use of radioactive reagents used in other in vitro tests and it permits the simultaneous quantitative measurement of specific IgE directed against 35 different allergens [24]. MAST-CLA sensitivity, specificity, and precision equal those of RAST and skin prick tests [25]. However, the MAST-CLA frequently shows concurrent positive results for multiple allergens in a single panel test; hence the clinicians can be confused as to whether the results should be interpreted as multiple allergens, cross-reactivity, or false positives. In this study, we used MAST-CLA results for detecting allergen-specific IgE antibodies, not for the determination of the specific allergens. Therefore, the definition of allergy in our study population was likely reliable. The criteria for the presence of allergy and the allergens affecting nasal allergy should be studied further, because current diagnostic tests provide evidence only of an increased amount of specific IgE antibody in the skin or blood serum, which cannot be related directly to nasal mucosa and Eustachian tube.

Another possible explanation for the negative correlation between the presence of allergy and OME is whether the middle ear mucosa is the target organ of the allergic inflammation. As mentioned above, the suggested mechanism of allergic involvement in OME is Eustachian dysfunction. However, not all allergic patients develop OME nor do all OME patients have allergies. Yeo et al. evaluated the relationship of serum and effusion fluid immunoglobulin concentrations, with the presence of bacteria in effusion fluid determined by culture and PCR, and found no correlation between the immunoglobulin concentration in middle ear effusion and the presence of bacteria in the effusion, while the serum immunoglobulin concentration was significantly related to the presence of effusion bacteria [26]. They suggested that the higher serum antibody concentration in bacteria-positive OME patients was due to a systemic immune reaction caused by inflammation in response to a local infection in the middle ear cavity. The relationship between systemic immune reaction and local infection in middle ear effusion should be evaluated in further studies.

Bacteria detection rates of up to 94.5 % have been reported in middle ear effusions using PCR; we observed a 70.4 % overall detection rate. The detection of bacterial DNA by PCR does not imply the existence of metabolically active bacteria, since PCR assays rely on the detection of genetic material regardless of the viability of the organism. The discrepancy between the low detection rate of the bacteria using conventional culture and the high detection rate using PCR can be explained by the use of antibiotics before myringotomy and the insertion of ventilating tubes, the involvement of biofilms in the progression of infection, and intracellular infection persistence of bacteria in the middle ear mucosa [10, 27, 28].

## Conclusion

Bacteria were found in more than 70 % of the middle ear effusions, with more than one third showing multiple bacteria. The bacteria detection rates did not differ between allergic and non-allergic children.

## Abbreviations

OME: otitis media with effusion
MEE: middle ear effusion
PCR: polymerase chain reaction
MAST: multiple allergosorbent test
RAST: radio-allergosorbent test

## References

[1] Kwon C, Lee HY, Kim MG, Boo SH, Yeo SG (2013). Allergic diseases in children with otitis media with effusion. International journal of pediatric otorhinolaryngology.

[2] Gok U, Bulut Y, Keles E, Yalcin S, Doymaz MZ (2001). Bacteriological and PCR analysis of clinical material aspirated from otitis media with effusions. International journal of pediatric otorhinolaryngology.

[3] Pelikan Z (2009). Role of nasal allergy in chronic secretory otitis media. Current allergy and asthma reports.

[4] Kariya S, Okano M, Hattori H, Sugata Y, Matsumoto R, Fukushima K (2006). TH1/TH2 and regulatory cytokines in adults with otitis media with effusion. Otology & neurotology : official publication of the American Otological Society, American Neurotology Society [and] European Academy of Otology and Neurotology.

[5] Lazo-Saenz JG, Galvan-Aguilera AA, Martinez-Ordaz VA, Velasco-Rodriguez VM, Nieves-Renteria A, Rincon-Castaneda C (2005). Eustachian tube dysfunction in allergic rhinitis. Otolaryngology--head and neck surgery : official journal of American Academy of Otolaryngology-Head and Neck Surgery.

[6] Bluestone CD, Stephenson JS, Martin LM (1992). Ten-year review of otitis media pathogens. Pediatr Infect Dis J.

[7] Blair C, Nelson M, Thompson K, Boonlayangoor S, Haney L, Gabr U (2001). Allergic inflammation enhances bacterial sinusitis in mice. The Journal of allergy and clinical immunology.

[8] Naclerio R, Blair C, Yu X, Won Y-S, Gabr U, Baroody FM (2006). Allergic rhinitis augments the response to a bacterial sinus infection in mice: A review of an animal model. American Journal of Rhinology.

[9] Park CW, Han JH, Jeong JH, Cho SH, Kang MJ, Tae K (2004). Detection rates of bacteria in chronic otitis media with effusion in children. Journal of Korean medical science.

[10] Coates H, Thornton R, Langlands J, Filion P, Keil AD, Vijayasekaran S (2008). The role of chronic infection in children with otitis media with effusion: evidence for intracellular persistence of bacteria. Otolaryngology--head and neck surgery : official journal of American Academy of Otolaryngology-Head and Neck Surgery.

[11] Labadie RF, Jewett BS, Hart CF, Prazma J, Pillsbury HC (1999). Allergy increases susceptibility to otitis media with effusion in a rat model. Second place--Resident Clinical Science Award 1998. Otolaryngology--head and neck surgery : official journal of American Academy of Otolaryngology-Head and Neck Surgery.

[12] Ebmeyer J, Furukawa M, Pak K, Ebmeyer U, Sudhoff H, Broide D (2005). Role of mast cells in otitis media. The Journal of allergy and clinical immunology.

[13] de Ru JA, Grote JJ (2004). Otitis media with effusion: disease or defense?. International journal of pediatric otorhinolaryngology.

[14] Kouwen H, van Balen FA, Dejonckere PH (2005). Functional tubal therapy for persistent otitis media with effusion in children: myth or evidence?. International journal of pediatric otorhinolaryngology.

[15] Revai K, Dobbs LA, Nair S, Patel JA, Grady JJ, Chonmaitree T (2007). Incidence of acute otitis media and sinusitis complicating upper respiratory tract infection: the effect of age. Pediatrics.

[16] Doyle WJ (2002). The link between allergic rhinitis and otitis media. Current opinion in allergy and clinical immunology.

[17] Tewfik TL, Mazer B (2006). The links between allergy and otitis media with effusion. Curr Opin Otolaryngol Head Neck Surg.

[18] Hurst DS (2008). Efficacy of allergy immunotherapy as a treatment for patients with chronic otitis media with effusion. International journal of pediatric otorhinolaryngology.

[19] Souter MA, Mills NA, Mahadevan M, Douglas G, Ellwood PE, Asher MI (2009). The prevalence of atopic symptoms in children with otitis media with effusion. Otolaryngology--head and neck surgery : official journal of American Academy of Otolaryngology-Head and Neck Surgery.

[20] Yeo SG, Park DC, Eun YG, Cha CI (2007). The role of allergic rhinitis in the development of otitis media with effusion: effect on eustachian tube function. American journal of otolaryngology.

[21] Chantzi FM, Kafetzis DA, Bairamis T, Avramidou C, Paleologou N, Grimani I (2006). IgE sensitization, respiratory allergy symptoms, and heritability independently increase the risk of otitis media with effusion. Allergy.

[22] Miceli Sopo S, Zorzi G, Calvani M (2004). Should we screen every child with otitis media with effusion for allergic rhinitis?. Arch Dis Child.

[23] Ogino S, Bessho K, Harada T, Irifune M, Matsunaga T (1993). Evaluation of allergen-specific IgE antibodies by MAST for the diagnosis of nasal allergy. Rhinology.

[24] Scolozzi R, Vicentini L, Boccafogli A, Camerani A, Pradella R, Cavallini A (1992). Comparative evaluation of RAST and MAST-CLA for six allergens for the diagnosis of inhalant allergic disease in 232 patients. Clin Exp Allergy.

[25] Finnerty JP, Summerell S, Holgate ST (1989). Relationship between skin-prick tests, the multiple allergosorbent test and symptoms of allergic disease. Clin Exp Allergy.

[26] Yeo SG, Park DC, Lee SK, Cha CI (2008). Relationship between effusion bacteria and concentrations of immunoglobulin in serum and effusion fluid in otitis media with effusion patients. International journal of pediatric otorhinolaryngology.

[27] Daniel M, Imtiaz-Umer S, Fergie N, Birchall JP, Bayston R (2012). Bacterial involvement in otitis media with effusion. International journal of pediatric otorhinolaryngology.

[28] Fergie N, Bayston R, Pearson JP, Birchall JP (2004). Is otitis media with effusion a biofilm infection?. Clin Otolaryngol Allied Sci.

