# Erratum to: Detection of bacteria in middle ear effusions based on the presence of allergy: does allergy augment bacterial infection in the middle ear?

**DOI:** 10.1186/s40463-016-0129-3

**Published:** 2016-02-19

**Authors:** Woo Jin Kim, Byung-Guk Kim, Ki-Hong Chang, Jeong-Hoon Oh

**Affiliations:** Department of Otorhinolaryngology-Head and Neck Surgery, College of Medicine, The Catholic University of Korea, Seoul, Korea; Department of Otorhinolaryngology-Head and Neck Surgery, The Catholic University of Korea, 180 Wangsan-Ro, Dongdaemun-Gu, Seoul, 130-709 South Korea

## Erratum

Unfortunately, the original version of this article [1] contained a paragraph in which the meaning was unclear.

The original paragraph read:

“This study demonstrates that the children with evidence of allergy were equally likely as children without evidence of allergy to demonstrate evidence of bacterial infection in PCR tested MEE specimens. Although this study did not demonstrate a positive relationship between allergy and the bacterial infection in the pathogenesis of OME. However, numerous studies have indicated that allergic subjects are more susceptible to OME than non-allergic controls.”

However this sentence should read:

“This study demonstrates that the MEE specimens from children with evidence of allergy were equally likely as those from children without evidence of allergy to demonstrate evidence of bacterial infection by PCR. Although this study did not demonstrate a positive relationship between allergy and the bacterial infection in the pathogenesis of OME, numerous studies have indicated that allergic subjects are more susceptible to OME than non-allergic controls.”
